# Correlation between full-thickness degenerative supraspinatus tear and radiographic parameters including the acromiohumeral centre edge angle and the greater tuberosity angle

**DOI:** 10.1186/s12891-021-04489-x

**Published:** 2021-07-06

**Authors:** Chaiyanun Vijittrakarnrung, Praman Fuangfa, Suphaneewan Jaovisidha, Chusak Kijkunasathian

**Affiliations:** 1 Department of Orthopedics, Faculty of Medicine Ramathibodi Hospital, Mahidol University, 270, Rama VI Road, Ratchathewi District, Bangkok, 10400 Thailand; 2 Department of Diagnostic and Therapeutic Radiology, Faculty of Medicine Ramathibodi Hospital, Mahidol University, 270, Rama VI Road, Ratchathewi District, Bangkok, 10400 Thailand

**Keywords:** Rotator cuff tear, Acromiohumeral centre edge angle, Greater tuberosity angle, Subacromial impingement, Radiographic measurement

## Abstract

**Background:**

Many radiographic parameters associated with the extrinsic cause of supraspinatus tears have been proposed. The aim of this study was to assess the relationship between a full-thickness degenerative supraspinatus tear (FTDST) and the patient’s radiographic parameters, including the acromiohumeral centre edge angle (ACEA) and the greater tuberosity angle (GTA).

**Methods:**

A retrospective study was conducted. We included 116 patients who underwent shoulder arthroscopic surgery at our institute. The case group included FTDST patients, whereas the control group also included patients without evidence of supraspinatus tears. In each patient, the ACEA and GTA values were measured and analyzed by two independent observers. Intra- and interobserver reliabilities were assessed. Multivariate regression analysis was performed.

**Results:**

The ACEA values were significantly increased in the FTDST group with a mean of 26.44° ± 9.83° compared with 16.81° ± 7.72° in the control group (*P* < 0.001). Multivariate regression analysis also showed that higher ACEA values were associated with an FTDST (odds ratio 1.16 per degree, *P* = 0.01). For GTA values, a statistically significant difference was found with a mean of 70.92° ± 6.64 compared with 67.84° ± 5.56 in the control group (*P* = 0.02). However, stepwise regression analysis did not indicate that GTA was a predictor of FTDST.

**Conclusions:**

Our study demonstrated that the presence of increased ACEA values is an independent significant risk factor for the presence of FTDSTs. Consequently, GTA values may be less helpful in assessing the risk of FTDST, especially in this specific population.

## Background

Rotator cuff tears (RCTs) are a common cause of chronic shoulder pain and disability [1]. Extrinsic causes for RCTs are usually associated with subacromial impingement [2], which is defined by the supraspinatus (SSP) tendon becoming entrapped between the acromion process and the greater tuberosity. Given that concern about SSP pathology has increased, many previous studies focused on excessive lateral acromial coverage and confirmed that it is associated with an increased incidence of RCTs [3–5]. Regardless, the complex interlinkage of these acromial morphology parameters with RCTs remains under extensive exploration.

Recently, Singleton et al. [6] introduced the acromiohumeral centre edge angle (ACEA), a new measurement to be used with true AP shoulder radiography. As a valid measurement with good reproducibility, the lateral projection of the acromion coverage humeral head exhibits an accuracy comparable to measurement using a computed tomography scan. The results showed that the ACEA value was significantly higher in patients with RCTs. However, because the Singleton et al. study only included acute traumatic RCTs, the usefulness of ACEA in predicting degenerative RCTs remains questionable. Furthermore, the subacromial impingement process necessarily comprises at least one side of the bony structure, making evaluation of both bony sides, the acromion, and the greater tuberosity (GT) equally important. GT morphology still plays an important role in subacromial impingement. Some studies report that a fracture malunion of GT was related to the worst outcome [7] and that the tuberosity procedure may provide a satisfactory result for irreparable massive rotator cuff tears [8]. Additionally, the greater tuberosity angle (GTA), a new radiographic marker that evaluates the GT position proposed by Cunningham et al., has been advocated as a reliable predictor marker for RCTs. Cunningham et al. suggested that the development of degenerative RCTs [9] was associated with higher GTA values. However, their study group included both participants with partial-thickness RCTs and participants with full-thickness RCTs. According to a recent study [10], some acromion parameters are associated with full-thickness but not with partial-thickness RCTs. Thus, the specific full-thickness degenerative supraspinatus tear (FTDST) subgroup correlation with these parameters should be determined.

No previous study has considered any of these values, including ACEA and GTA, as a specific tool to be used with patients with FTDST. Moreover, the literature contains limited information about these parameters, especially those pertaining to a Southeast Asian population. Thus, we attempted to evaluate the correlation of both parameters to the incidence of FTDST and analyze any association of both parameters with patients’ demographic data and arthroscopic findings. The primary objectives of our study were [1] to evaluate the presence of significant differences regarding the ACEA and GTA values among patients with or without FTDST and [2] to assess the association between any of these parameters and other variables, such as age, sex, and SSP tear retraction. The hypothesis was that higher values for ACEA and GTA would be associated with a greater likelihood of detecting FTDST.

## Materials and methods

### Sample

A retrospective study was conducted in Ramathibodi Hospital, Mahidol University, Thailand. The medical records of all patients who underwent shoulder arthroscopic surgery between April 2016 and July 2018 were collected. All preoperative true AP shoulder radiographs were analyzed. The patients selected were divided into two groups. The case group consisted of individuals presenting with a clinical diagnosis of FTDST, which was confirmed by history, preoperative magnetic resonance imaging (MRI) scans, and intraoperative arthroscopic findings. The control group consisted of those with shoulder pain without any RCT findings based on history; physical examination; preoperative MRI scans; and arthroscopic findings, such as labral injury, shoulder instability, and primary adhesive capsulitis. Patient demographic data included age, sex, bicep pathology, fatty degeneration of the SSP by Goutallier staging [11], and SSP tear retraction in the coronal plane grading by Patte classification [12].

### Inclusion criteria & exclusion criteria

The inclusion criteria were that the patients had a preoperative MRI scan and a preoperative true AP shoulder radiograph with the proximal humerus in an acceptable rotation of the affected shoulder. Our target population age was 18–85 years old. The studied participants were recruited exclusively from the Thai population. The exclusion criteria included partial-thickness RCTs, any history of traumatic events to exclude any potential traumatic etiology, previous surgery, fractures, and/or dislocation around the shoulder, congenital shoulder deformity, shoulder tumors, or infection. Patients with any evidence of osteoarthritis change in the glenohumeral joint, including superior humeral head migration, were also excluded due to the possibility of producing outliers of these parameters.

Patients meeting the inclusion and exclusion criteria were recruited. Therefore, the final sample comprised 116 patients who were enrolled in the study. The case group included 84 patients, and the control group included 32 patients. This study was ethically approved by our hospital’s institutional research board committee (IRB number MURA2018/837). All methods in the study were performed in accordance with the Helsinki guidelines and relevant CIOMS guidelines.

### Data collection & outcome measurement

Data on fatty degeneration of the SSP by Goutallier staging and SSP tear retraction in the coronal plane grading by Patte classification were collected from preoperative MRI interpretation. Associate subscapularis (SSC) lesions and bicep pathology were assessed from both preoperative MRI interpretation and arthroscopic findings. In case of a mismatch between MRI findings and arthroscopic findings, we decided to accept an arthroscopic finding as a first priority.

**Fig. 1:**
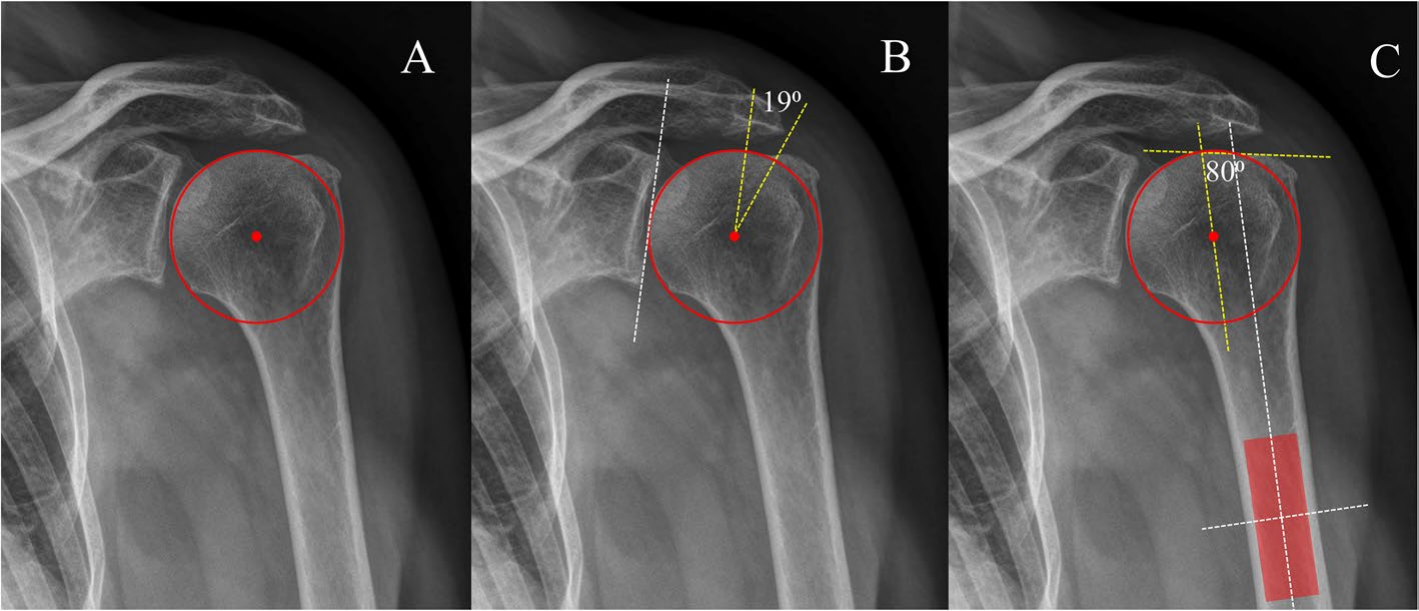
On a true AP glenohumeral X-ray, **A** the humeral head is circled, and the center of rotation is marked. **B** Measuring the ACEA formed with the first drawn superiorly from the center of the humerus parallel to the glenoid surface and the second drawn from the center of the circle to the lateral-most aspect of the acromion edge. **C** Measuring the GTA angle formed by the first drawn parallel to the diaphyseal axis that passes through the humeral head center of rotation; the second drawn connects the superior border of the humeral head to the superolateral edge of the greater tuberosity

ACEA and GTA measurements were determined on true AP shoulder radiographs and described, as shown in Fig. 1. The ACEA is defined as the angle between a line drawn superiorly from the center of the humeral head parallel to the glenoid and a line from the center of the humeral head to the acromion’s outer edge (Fig. 1B) [6]. The GTA represents the angle between a line parallel to the humerus diaphysis passing through the center of rotation of the humeral head and a line connecting the upper edge of the humeral head to the most superolateral edge of the GT (Fig. 1C) [9]. All measurements were performed by two independent assessors: an orthopedic surgeon specializing in the shoulder and a radiologist specializing in musculoskeletal imaging) using a goniometer tool in the Picture Archiving and Communication System (PACS). Both were blinded from intraoperative findings. Then, a repeated measurement was performed with a one-month interval. Interobserver and intraobserver reproducibility was determined.

In an effort to avoid the effect of rotation of the proximal humerus, the parameters were measured on the true AP shoulder radiograph. Each patient was placed in a supine position with the arm adducted to the side and in a neutral position. Rotation in the axial plane was accepted up to ± 20^0^. This protocol was performed with unchanged ACEA and GTA parameters in this rotation range according to previous studies [6, 9].

### Statistical analysis

Statistical analyses were calculated using Stata 16 software (StataCorp, College Station, TX, USA). The difference in the ACEA and GTA of the case and control groups was determined using independent t-tests. Correlations between parameters and age as well as the relationship between both measurements were assessed with the Pearson correlation coefficient method. Receiver operating characteristic (ROC) analysis curves were devised to determine the diagnostic ability of these parameters. Multivariate adjusted analysis by logistic regression analysis was used to determine each factor for the occurrence of FTDSTs with respective odds ratios. A Bonferroni post hoc test was performed to evaluate whether any significant differences existed among the subgroup analyses based on the SSP tear retraction grading. The limits of agreement between the two assessors were examined with Bland–Altman plot analysis. The intrarater reliability and interrater reliability were assessed using the intraclass correlation coefficient (ICC). The ICC was interpreted as follows: 0 to 0.40, poor; 0.41 to 0.60, moderate; 0.61 to 0.80, good; and 0.81 to 1.00, excellent [13].

## Results

### Patient demographic data

The total number of recruited samples was 116 shoulders. The case group included 84 shoulders representing shoulders with FTDST, whereas the control group (without evidence of SSP tear) included 32 shoulders. The mean age was 64.19 ± 7.67 years (range, 46–81 years) for the case group and 35.81 ± 14.13 years (range, 19–65 years) for the control group.

In every shoulder, we identified the presence of an SSP tear, SSP tear retraction, and biceps pathology. In our series, 61.9% had biceps pathology in the case group compared to 12.5% in the control group. There were 42 patients with SSP retraction grade 1 (50%), 33 patients with SSP retraction grade 2 (39%), and 7 patients with SSP retraction grade 3 (8%). All patient characteristics are listed in Table 1.

**Table 1:** Patient demographic characteristics

Demographic characteristics	Study population	
	**Case group (n = 84)**	**Control group (n = 32)**
**Age (years)**^a^		
Mean ± SD	64.19 ± 7.67	35.81 ± 14.13
**Gender**^b^		
Male	33 (39.3%)	23 (71.9%)
Female	51 (60.7%)	9 (28.1%)
**Side**^b^		
Right	58 (69%)	17 (53.1%)
Left	26 (31%)	15 (46.9%)
**Goutallier **[11]** classification**^b^		
Grade 0	20 (23.81%)	32 (100%)
Grade 1	51 (60.71%)	0 (0%)
Grade 2	12 (14.29%)	0 (0%)
Grade 3	1 (1.19%)	0 (0%)
**Patte **[12]** classification**^b^		
Normal	0 (0%)	32 (100%)
Grade 1	42 (50%)	0 (0%)
Grade 2	33 (39.28%)	0 (0%)
Grade 3	7 (8.33%)	0 (0%)
**Bicep pathology**^b^	52 (61.9%)	4 (12.5%)
**Biglini acromial types**^b^		
Type I (Flat)	29 (34.5%)	26 (81.2%)
Type II (Curve)	42 (50%)	6 (18.8%)
Type III (Hook)	13 (15.5%)	0 (0%)
**SSC **[14]** pathology**^b^		
No injury	15 (17.86%)	32 (100%)
Type I	15 (17.86%)	0 (0%)
Type II	40 (47.62%)	0 (0%)
Type III	12 (14.29%)	0 (0%)
Type IV	2 (2.38%)	0 (0%)
Type V	0 (0%)	0 (0%)
**Pathology**^b^		
FTDST	84 (100%)	
Bankart lesion		14 (43.75%)
SLAP lesion		
Type II		2 (6.25%)
Type V		4 (12.5%)
Adhesive capsulitis		12 (37.5%)

^a^: value presented as the mean ± standard deviation

^b^: value presented as the number of volunteers with that condition (percentage)

### Radiographic interpretation

The ACEA and GTA were accessible in 116 shoulders. The average ACEA value was 23.79° ± 10.22°, and the average GTA value was 70.07° ± 6.49°. Comparing both parameters with the presence of FTDST, we found that both angles had a statistically significant association with the presence of SSP tears. The means of the ACEA and GTA variables in the case group were 26.44° ± 9.83° (95% CI, 24.31°–28.57°) and 70.92° ± 6.64 (95% CI, 69.48°–72.36°), respectively. In the control group, the means of the ACEA and GTA variables were 16.81° ± 7.72° (95% CI, 14.03°–19.60°) and 67.84° ± 5.56 (95% CI, 65.84°–69.85°), respectively. Statistically significant associations between both variables and FTDST were found as shown in Table 2.

**Table 2:** Comparison of the parameters between patients with or without full-thickness degenerative supraspinatus tears (FTDSTs)

Parameters	Group	Statistics			
		**N**	**Mean (°)**	**SD (°)**	***p-*value**
**ACEA**	Case group	84	26.44	9.83	< 0.001**
	Control group	32	16.81	7.72	
**GTA**	Case group	84	70.92	6.64	0.02*
	Control group	32	67.84	5.56	

^*^Significant at level 0.05

^**^Significant at level 0.01

For the assessment of any correlation between age and these parameters among total populations, a small positive strength of association (coefficient < 0.3) was found between age and both parameters. However, the results showed no significant differences between these parameters within each group as noted in Table 3.

**Table 3:** Correlation analysis between parameters and age in the total population and in each group

Parameters	Total (n = 116)		Case group (n = 84)		Control group (n = 32)	
	**Pearson correlation**	***p-*value**	**Pearson correlation**	***p-*value**	**Pearson correlation**	***p-*value**
**ACEA**	0.29	0.001**	-0.22	0.045	0.18	0.324
**GTA**	0.25	0.006**	0.27	0.014	-0.05	0.772

^**^Significant at level 0.01

Comparing both parameters in correlation with patient gender, Table 4 shows that females had a significantly higher ACEA value than males in the total population. No statistically significant difference in the GTA value was noted based on gender.

**Table 4:** Comparison of parameters by gender among the total population and in each of both groups

Parameters	Total (n = 116)				Case group (n = 84)				Control group (n = 32)			
	**N**	**Mean (°)**	**SD (°)**	***p-*value**	**N**	**Mean (°)**	**SD (°)**	***p-*value**	**N**	**Mean (°)**	**SD (°)**	***p-*value**
**ACEA**												
Female	60	25.83	10.78	0.025**	51	27.24	10.69	0.36	9	17.89	7.69	0.63
Male	56	21.59	9.18		33	25.21	8.33		23	16.39	7.87	
**GTA**												
Female	60	70.88	7.31	0.16	51	71.08	7.6	0.783	9	69.78	5.59	0.224
Male	56	69.19	5.4		33	70.67	4.89		23	67.09	5.49	

^**^Significant at level 0.01

The ROC curves were designed to evaluate the ability of both angles to predict FTDST.

**Fig. 2:**
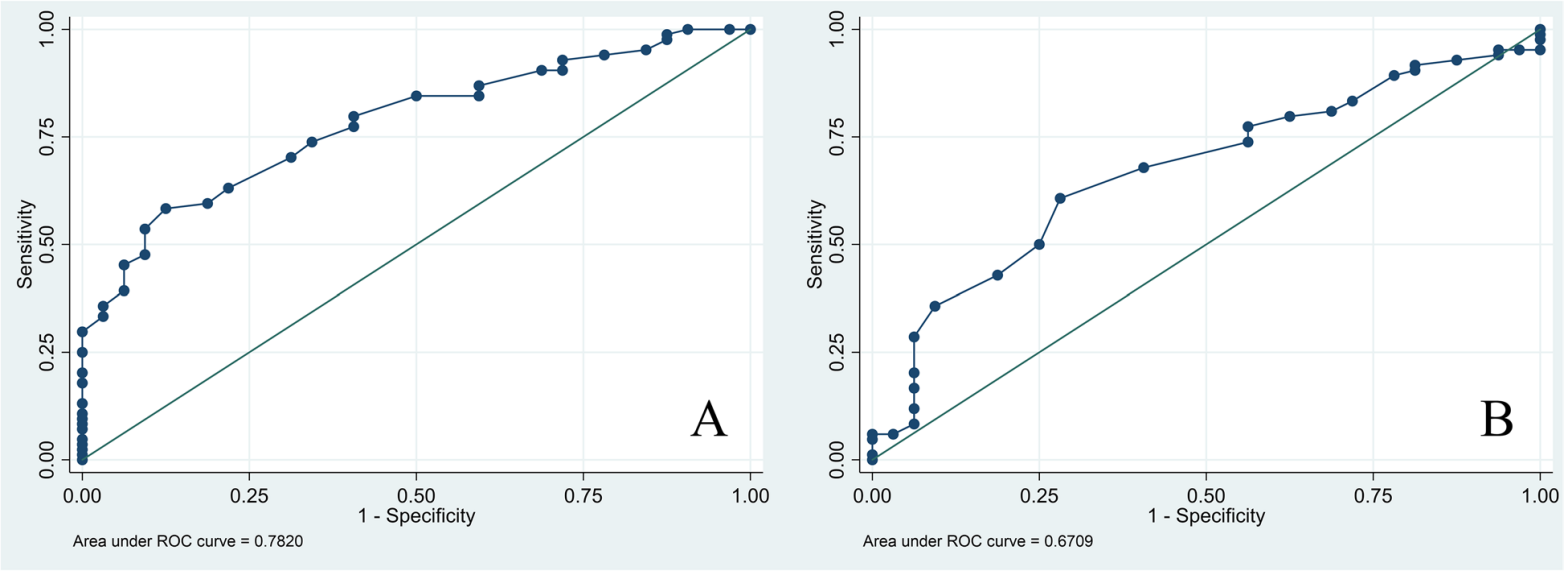
**A** ACEA, **B** GTA. Receiver operating characteristic (ROC) curve with areas under the ROC curve of 0.78 and 0.67, respectively

The curves showed that an ACEA was a good predictor of FTDST with an area under the curve of 0.78. For a GTA value, the area under the curve was 0.67, which is interpreted as a fair predictor of FTDST (Fig. 2). To determine the cut point, an ACEA value of 18° was a good predictor of full-thickness SSP tears with 85% sensitivity and 50% specificity. In contrast, a GTA value of 68° had 77% sensitivity and 44% specificity. This finding indicated that the ACEA value is a more accurate diagnostic test than the GTA value. The differences in the cutoff values of ACEA and GTA are reported in Table 5.

**Table 5:** Different ACEA and GTA cutoff values (PPV: positive predictive value, NPV: negative predictive value)

Cutoff value	Sensitivity (%)	Specificity (%)	PPV (%)	NPV (%)	Accuracy (%)
**ACEA**					
15°	90.5	31.3	77.6	55.6	74
16°	86.9	40.6	79.3	54.2	74
17°	84.5	40.6	78.9	50.0	72
18°	84.5	50.0	81.6	55.2	75
19°	79.8	59.4	83.8	52.8	74
20°	77.4	59.4	83.3	50.0	72
21°	73.8	65.6	84.9	28.8	72
**GTA**					
65°	83.3	28.1	75.3	39.1	68
66°	81.0	31.3	75.6	38.5	68
67°	79.8	37.5	77.0	41.4	68
68°	77.4	43.8	78.3	42.4	67
69°	73.8	43.8	77.5	38.9	66
70°	67.9	59.4	81.4	41.3	66
71°	60.7	71.9	85.0	41.1	64

**Fig. 3:**
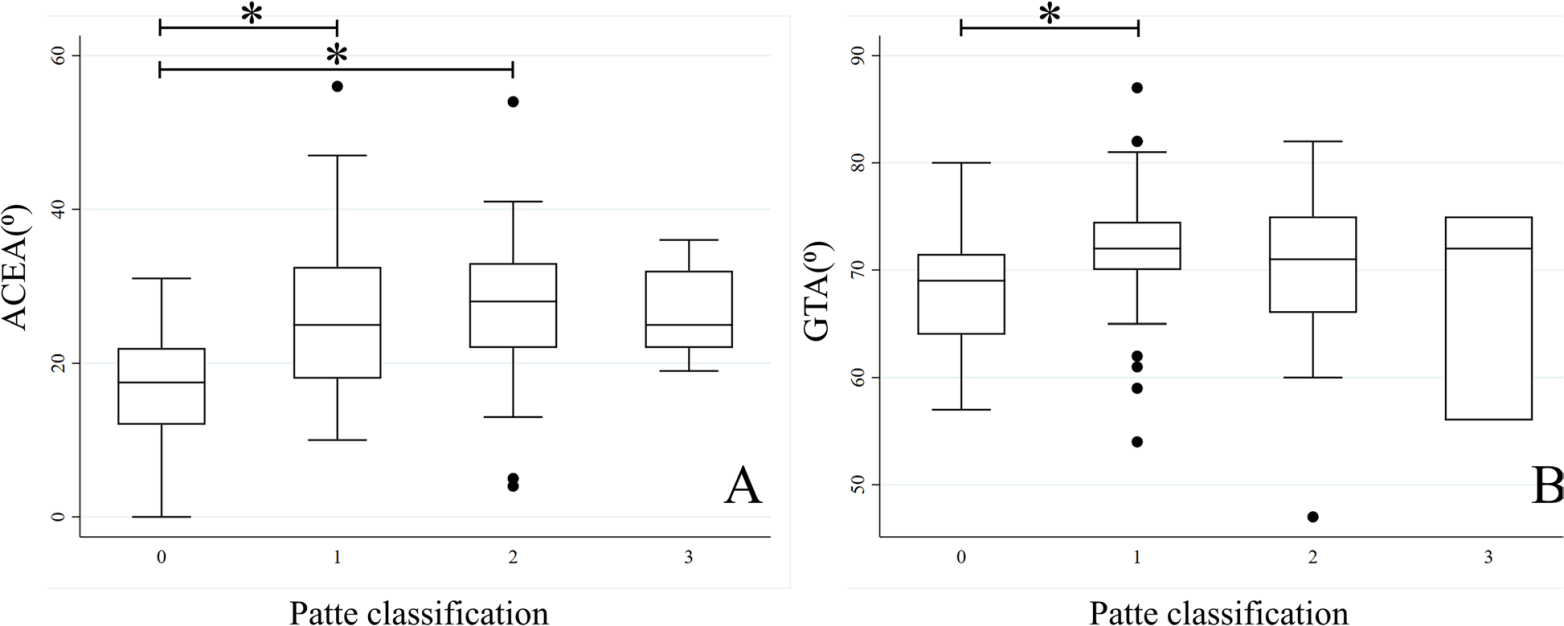
Comparison of parameters. **A** ACEA and **B** GTA between the groups graded by Patte classification. Error bars indicate the interquartile range (IQR) of the median. Black dots indicate values above the upper fence (1.5*IQR). * above the lines spanning between groups indicates *P-*values < 0.05. Grade 0 indicates no rotator cuff tear condition

The multivariate analysis showed that the ACEA parameter was the only parameter that was found to be statistically significant. A higher ACEA value indicated an increased risk of FTDST with an odds ratio of 1.16 per degree (*P* = 0.01). However, no statistical significance was noted for the GTA parameter (*P* = 0.10) (Table 6). The risk factor for FTDST was increased age (odds ratio of 1.26 per year; *P* < 0.001). Our findings also showed that while the mean ACEA and GTA values of the FTDST group were greater than those of the control group, the means of the parameters among subgroups categorized by Patte classification did not show a significant difference. A comparison of both parameters is shown in Fig. 3.

**Table 6:** Multivariate analysis by logistic regression analysis for each factor associated with the presence of full-thickness degenerative supraspinatus tears (FTDSTs)

Factor	Odd ratio	95% Confidence interval		*p-*value
ACEA, per degree	1.16	1.04	-1.3	0.01**
GTA, per degree	1.13	0.98	-1.32	0.10
Age, per year	1.26	1.13	-1.43	< 0.001**
Gender, female to male	0.92	0.17	-5.01	0.93

^**^Significant at level 0.01

Pearson's product-moment correlation was performed to assess the relationship between ACEA and GTA. Only a negligible negative correlation was noted between ACEA and GTA in the case group (*r* = -0.238, *P* = 0.03); however, the results showed no significant differences between another comparison (total population, control group) as noted in Table 7.

**Table 7:** Correlation analysis between both parameters in the total population and in each group

Parameters	Total (n = 116)		Case group (n = 84)		Control group (n = 32)	
	**Pearson correlation**	***p-*value**	**Pearson correlation**	***p-*value**	**Pearson correlation**	***p-*value**
**ACEA—GTA**	-0.069	0.46	-0.238	0.03*	0.06	0.76

^*^Significant at level 0.05

**Fig. 4:**
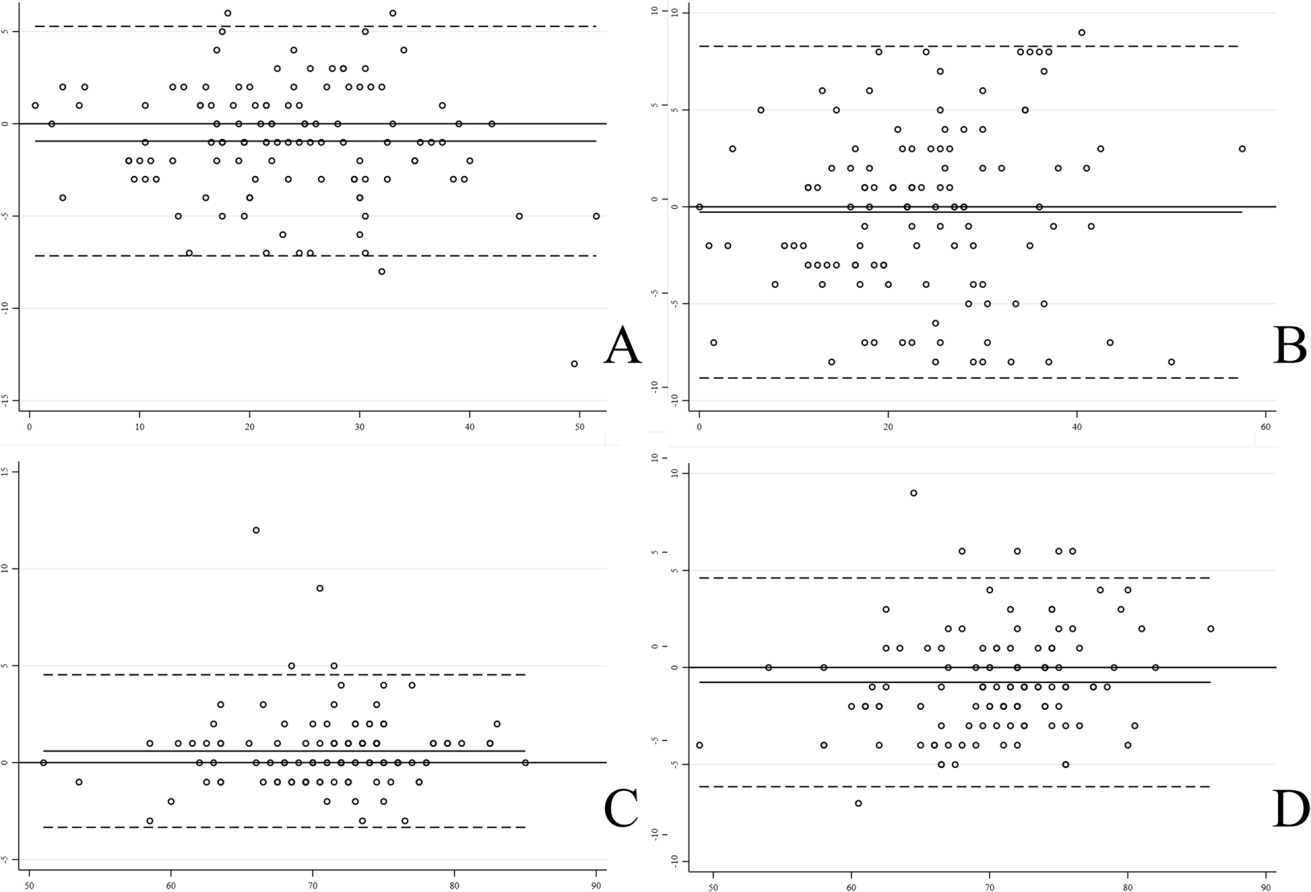
Bland–Altman plot of the mean difference in ACEA measurements between one-month separate time points (**A**). The mean difference in ACEA measurements between the two assessors (**B**). The mean difference between GTA measurements taken at one-month separate time points (**C**). The mean difference between GTA measurements of two assessors (**D**)

Reliability testing for the ACEA and GTA values showed that the mean ACEA difference and the mean GTA difference were -0.94 ± 3.2 and 0.60 ± 2.0, respectively. The ICC values for the ACEA and GTA measurements were 0.95 and 0.94, respectively. Interobserver reproducibility for the ACEA and GTA values showed that the mean ACEA difference and the mean GTA difference between both assessors were -0.27 ± 4.37 and -0.77 ± 2.75, respectively. The ICC values for the ACEA and GTA measurements between both assessors were 0.91 and 0.89, respectively (Table 8). The Bland–Altman plot of the mean difference between the repeated measurements is shown in Fig. 4.

**Table 8:** Summary of intrarater and interrater reliability of ACEA and GTA (LOA: limits of agreement, ICC: intraclass correlation coefficient)

Parameters	Intraobserver reliability			Interobserver reliability		
	Mean ± SD (°)	95% LOA (°)	ICC (%)	Mean ± SD (°)	95% LOA (°)	ICC (%)
ACEA	-0.94 ± 3.2	-7.16 to 5.27	95	-0.27 ± 4.37	-8.84 to 8.283	91
GTA	0.60 ± 2.0	-3.34 to 4.53	94	-0.77 ± 2.75	-6.15 to 4.61	89

## Discussion

RCTs are one of the most common causes of chronic shoulder pain, leading to decreased functionality, decreased quality of life and increased utilization of health care resources [15]. Due to its cost effectiveness and accessibility, the standard shoulder series is typically the first line of investigation for patients suspected of having RCTs [16] to provide additional information and the need for advanced images, such as MRI of the shoulder [17]. This study aims to correlate radiographic parameters, such as ACEA and GTA, from standard shoulder radiographs and FTDST in patients who presented with shoulder pain who underwent arthroscopic surgery.

Our results showed that the mean ACEA parameter was 26.44° ± 9.83° in the case group compared to 16.81° ± 7.72° in the control group, and a statistically significant difference was noted between the groups. Females had a significantly higher ACEA value than males in the total population. Pearson correlation was used to identify any correlations present between age and ACEA among this study’s total population. We found a negligible correlation between the ACEA value and age [18]. According to logistic regression analysis, an increased ACEA has an independent risk of FTDST with an odds ratio of 1.16 per degree. In addition to higher ACEA values, the analysis showed that increased age was a risk factor in the FTDST. Our results regarding patient age aligned with those of prior studies, which revealed that the prevalence of RCTs positively correlated with patient age [1]. The results of the present study with FTDST are comparable with those in the study by Singleton et al., which reported a positive correlation between ACEA and acute traumatic RCT (23.9 vs. 16.6, *P* < 0.001) [6]. However, they did not consider RCT size and mentioned that implementation of the ACEA parameter on chronic degenerative tears remains doubtful. Based on our results, we certified that ACEA could be generalized to an FTDST population and used as a reliable measurement tool for detecting FTDST on standard shoulder plain radiographs. The association between higher ACEA values and rotator cuff pathology could be clarified in the same way that it has been for other parameters, such as the acromial index (AI) and critical shoulder angle (CSA). This finding was explained by the vertical force vector of the middle fiber of the deltoid muscle, where the pull influenced by the lateral extension of the acromion was directed upward. These effects potentially led to SSP impingement and a consequent tear due to a compression effect causing a degeneration tear of the rotator cuff as stated in previous literature [19, 20].

Despite the high variability of GT morphology [21], the GTA parameter was first proposed in 2018 as a new reliable radiographic marker of degenerative RCTs. However, the precise relationship between high GTA values and the incidence of RCTs is not yet understood. In the present study, GTA significantly differed between groups with a small size difference; the mean in the case group was 70.92° ± 6.64 compared to 67.84° ± 5.56 in the control group. However, multivariable analysis showed no statistically significant correlation between GTA and FTDST. Hence, stepwise multiple regression analysis rejected GTA as a predictor of FTDST. The results of the present study contrast with those reported by Cunningham et al., who noted that the mean GTA parameter was 72.5° ± 2.5° in the RCT group compared to 65.2° ± 4.1° in the control group and concluded that degenerative RCTs in the European population were associated with GTA values of greater than 70°[9]. In addition, Yoo et al. reported that high GTA values accompanied RCTs in the Korean population based on MRI results [22]. This discrepancy between results could be explained by the population-based anatomic variation that may exist in a Southeast Asian population, such as the Thai population. A recent study documented that the Asian population exhibits a smaller humeral head than the Western population [23]; these findings could possibly explain why the GTA effect is greater in Asians compared with Europeans [22]. Moreover, previous studies examined the GTA effect on individuals with partial- or full-thickness supraspinatus tears. However, our study examined the GTA effect only on FTDST; partial tears were not taken into consideration. Recent studies [5, 10] demonstrated a dissimilarity association between acromial parameters and the type of RCT tear (full thickness vs. partial thickness). Additionally, Seo et al. [24] reported that the mean GTA values for bursal-side partial RCTs tended to be greater than those for full-thickness RCTs. Therefore, the different population characteristics could affect the magnitude of the parameter and may have produced different findings among studies as well.

This study was the first to evaluate these angles based on arthroscopic findings for their usefulness in determining the risk of FTDST. The most important finding was that ACEA exhibited a statistically significant, high association with FTDST. Stepwise logistic regression analysis demonstrated that between ACEA and GTA, only ACEA appears to exhibit a valid correlation with FTDST. GTA was rejected as a factor in predicting FTDST. No previous study has performed logistic regression to assess the effects of these parameters on FTDST. We summarized that ACEA could be used as an independent factor to assess the risk of FTDST in our study. Thus, we also concluded that GTA could not be utilized to assess the risk of FTDST in the Southeast Asian population, particularly in the Thai population. Nevertheless, a positive correlation was not found between a higher value and SSP tear retraction grading. Thus, these parameters cannot be used to differentiate the severity of tear retraction of SSP. Although the possibility of type II errors should be considered, further biomechanics studies are essential for specific assessment of the influence of these parameters on tear retraction.

To apply these findings to patient management, our study revealed that these angle measurements would provide additional information and the needs of further advanced images, such as MRI, to diagnostic urgency conditions, which usually require operative treatment, such as FTDST [25]. This study chooses to evaluate the ACEA and GTA. The first parameter represents lateral acromial extension for humeral head coverage. The latter represents the importance of GT prominence related to decreased distance between acromion and GT. Both of these angle measurements relied on the same center rotation of the humeral head, which led to more straightforward steps and is less prone to error measurement. Furthermore, for stability according to humerus rotation, these measurements remained unchanged in the acceptable range of rotation in the axial plane [6, 9] in contrast to other measurements, such as AI and CSA, which might potentially affect diagnostic accuracy due to this rotation variation [26].

The present study had several limitations. First, due to the limitations of the arthroscopic-based study design and retrospective nature of the study, the control group didn’t include normal healthy participants. Since the control group was designed for those patients who underwent shoulder arthroscopic surgery from various pathologies, except for RCT condition. Correspondingly, some demographic data might have a difference between the groups. FTDST patients tend to be females who are older than patients with labral injury and shoulder instability, even adhesive capsulitis, which is mainly included in the control group; thus, age and sex matching was not optimized. Despite this variation, a statistically significant difference still existed for the ACEA parameter after multivariable analysis. Second, our study has a relatively small sample size compared to previous studies. However, a statistical power analysis was performed for sample size estimation with alpha = 0.05 and power = 0.80. The sample size needed with this effect size was approximately 25 samples within each group. This size was sufficient for detecting a difference of 5° between groups if the standard deviation of each group was 7.7. Thus, our proposed sample size was more than adequate for this study’s primary objective. Third, the difference in ACEA and GTA between the patients with and without FTDST was approximately 9.6° and 3.1°, respectively; these results were within the SD range. Although the findings were statistically significant, careful interpretation is essential to determine the clinical application of these parameters. Fourth, for the reason that superior migration and advanced osteoarthritis change of the proximal humerus might substantially alter these parameters' values, we meticulously excluded these changes from our study. Therefore, attentive interpretation should be considered in a patient with such alterations. Fifth, FTDSTs were not quantified or subcategorized into focal versus complete full-thickness tears. Finally, other acromion morphology, such as acromial down slopping was not investigated in this study.

## Conclusion

The presence of higher ACEA values is a significant independent risk factor for the presence of full-thickness, degenerative SSP tears. Among chronic shoulder pain patients who suspected a full-thickness, degenerative SSP tear condition from history and physical examination, the measurement of ACEA could represent a useful tool to determine additional advanced images, such as MRI, to confirm the diagnosis of this condition in this specific patient group. Consequently, GTA values may be less helpful in assessing the risk of full-thickness, degenerative SSP tears, especially in the Southeast Asian population.

## Data Availability

The datasets generated and/or analysed during the current study are not publicly available due to limitations of ethical approval involving the patient data and anonymity but are available from the corresponding author on reasonable request.

## Abbreviations

FTDST: Full-thickness degenerative supraspinatus tear
RCT: Rotator cuff tear
SSP: Supraspinatus
GT: Greater tuberosity
ACEA: Acromiohumeral centre edge angle
GTA: Greater tuberosity angle
MRI: Magnetic resonance imaging
MVA: Multivariate regression analysis
ICC: Intraclass correlation coefficient
CSA: Critical shoulder angle
AI: Acromion index
